# Temporal bone osteoblastoma involving temporomandibular joint diagnosed as simple disc disorders: A case report

**DOI:** 10.3389/fsurg.2022.1033342

**Published:** 2023-01-06

**Authors:** Feiya Zhao, Xinyue Zhang, Qiaoting Pan, Xin Ye, Mengfei Yu, Zhiyong Li, Huiming Wang

**Affiliations:** Stomatology Hospital, School of Stomatology, Zhejiang University School of Medicine, Zhejiang Provincial Clinical Research Center for Oral Diseases, Key Laboratory of Oral Biomedical Research of Zhejiang Province, Cancer center of Zhejiang University, Hangzhou, China

**Keywords:** osteoblastoma, temporomandibular joint, ossifying fibroma, temporomandibular joint disorder, osteoid osteoma, osteosarcoma

## Abstract

**Background:**

Osteoblastoma is quite rare in the oromaxillo-facial region, while the mandible is always the predilection. However, in our case, the lesion was located in the left temporal articular tubercle, involving the adjacent skull base, which is extremely rare in the literature.

**Case reports:**

It had been diagnosed as the most common temporomandibular joint disorder in the local hospital before the patient came to our department, mainly due to the primary symptom, that was, the patient got pain in the left temporomandibular joint area while opening the mouth. However, we found a mass of bone lesions at the left temporal articular tubercle in MRI and cone beam CT, and it turned out to be an osteoblastoma after surgery. The patient's primary symptom disappeared after recovering from the surgery, and there have been no indications of complication or recurrence up to now.

**Conclusion:**

Osteoblastoma is very rare in the temporomandibular joint region. It could easily miss the possibility of a benign tumor due to its unusual location and confusing chief complaint in this case. Our report provides experience in the identification of osteoblastoma in rare sites.

## Introduction

Osteoblastoma is a benign bone-forming tumor with a peak incidence in the second decade of life (1, 2), and its most common locations are the spine and long bones. It is quite rare in the stomatology department, and it tends to occur in the posterior mandible (3–6). In this case, the lesion was located in the left temporal bone, involving the temporomandibular joint (TMJ) and adjacent skull base, which is extremely rare in the literature. Osteoblastoma has two subtypes: the conventional/benign one and the osteoblastic/aggressive one (7). They look almost the same radiologically, but there are subtle differences in histopathology between them; that is, large epithelioid osteoblasts can be found in the latter one. Most importantly, the latter one is more aggressive and has a higher recurrence rate of about 15%–20% (8–10). Luckily, the lesion in this case was classified as a conventional osteoblastoma. However, it was reported that osteoblastoma located near important tissues has a poorer outcome after surgery, probably due to the difficulty associated with complete resection (1, 2, 6, 11), which indicates that the case we presented may have a higher recurrence.

The patient's clinical symptoms were quite similar to those of the most common temporomandibular joint disorders (TMD), and they may easily miss the possibility of a benign tumor. We therefore hope that this case report will provide experience in the identification of osteoblastoma in rare sites.

### Clinical presentation

**Figure 1:**
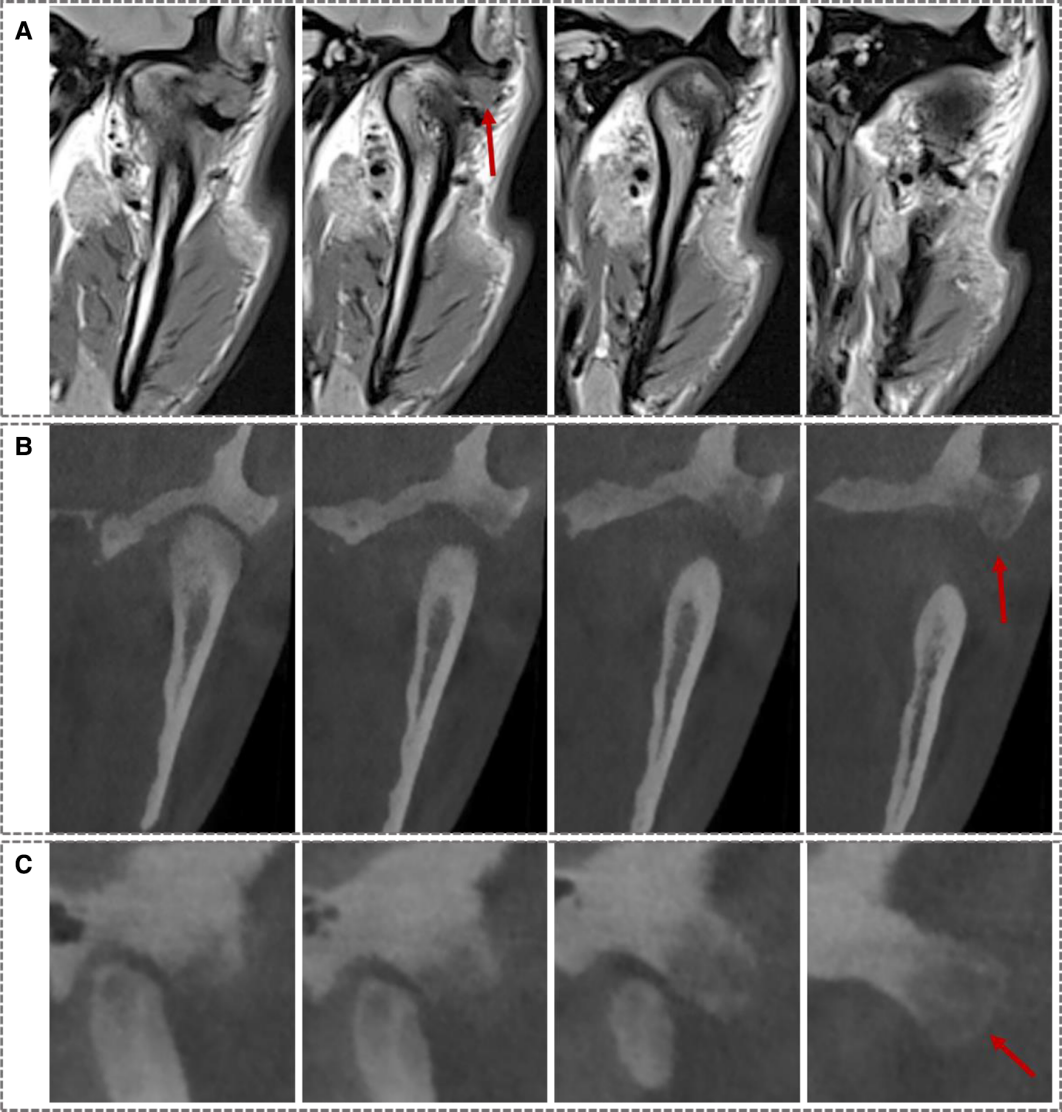
Preoperative MRI and CT images: (A) coronal MRI images showed a mass at the left articular tubercle. (B) Coronal CT images showed a heterogeneous mass at the left articular tubercle. (C) Sagittal CT images showed the same. (Red arrows indicates the lesion).

A 26-year-old male patient came to our department with the chief complaint of pain in the left preauricular area while opening his mouth for the last 3 years. It was diagnosed as TMD after a physical and radiological examination at the local hospital over a year ago. Besides giving instructions about protecting the TMJ area, “glucosamine hydrochloride capsule” was also given at that time to reduce intra-articular inflammation and prevent damage to the articular cartilage. However, the symptoms did not receive effective relief. Physical examination showed that there was swelling and pain with palpation in the left TMJ area. The largest mouth opening range was normal but with pain and deviation to the left, while without joint noise or limitation during protrusive and lateral sliding movements. There were no ear complaints, such as ear pain or hearing impairment. Then further radiological examinations were performed, including cone beam CT (CBCT) and magnetic resonance imaging (MRI), which showed anterior displacement of the left disc, and local swelling was detected on the anterior and lateral sides of the left temporal articular tubercle, with interrupted continuity of the cortical bone (Figure 1A). CBCT showed a heterogeneous mass sized approximately 14.5 mm * 10 mm * 8 mm at the left temporal articular tubercle (Figures 1B,C).

### Treatment and diagnosis

**Figure 2:**
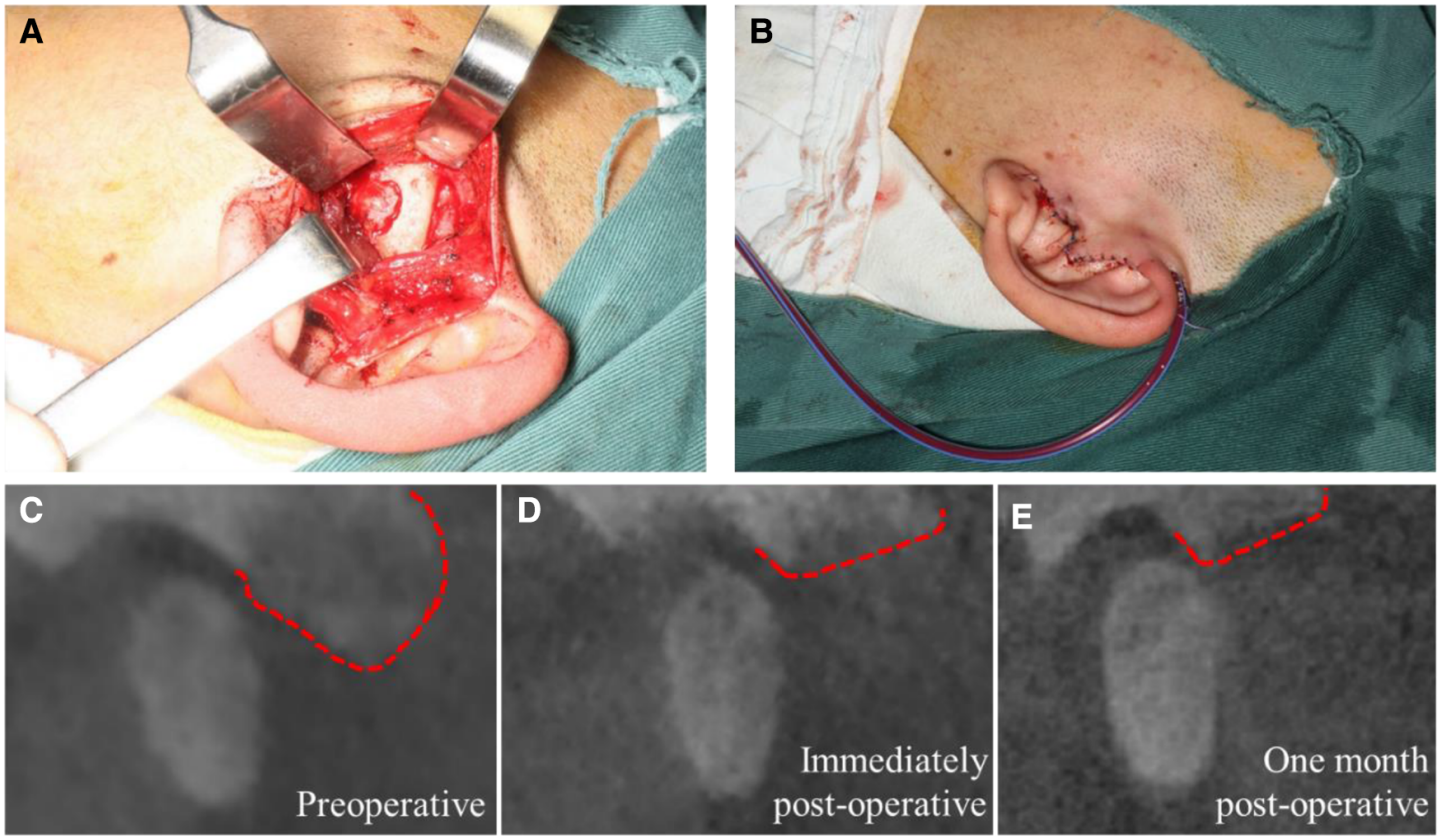
Surgical intervention and radiological change in CBCT images: (A) lesion resection at the left articular tubercle; (B) stitches of the incision and high vacuum drainage device placement; (C–E) CBCT images of the lesion area preoperatively, immediately postoperatively and one month postoperatively, respectively. (Red dotted lines show the border the articular tubercle region).

During the operation, a pre-auricular incision was made, the tumor was resected (Figure 2A), followed by curetting to the area with relatively normal bone, reshaping the articular eminence, disc manipulation was not performed, and finally, suturing the incision carefully and placing a high vacuum drainage device (Figure 2B). The intraoperative frozen biopsy could not be performed because the tissue was too hard to slice up immediately. Then all three masses resected or curetted (sized 1.1 cm × 0.8 cm × 0.5 cm, 0.3 cm × 0.3 cm × 0.2 cm, and 0.2 cm × 0.1 cm × 0.1 cm, respectively) were sent for histopathological examination. Immediately post-operative CBCT (Figure 2D) revealed the resection range compared with that pre-operatively (Figure 2C).

**Figure 3:**
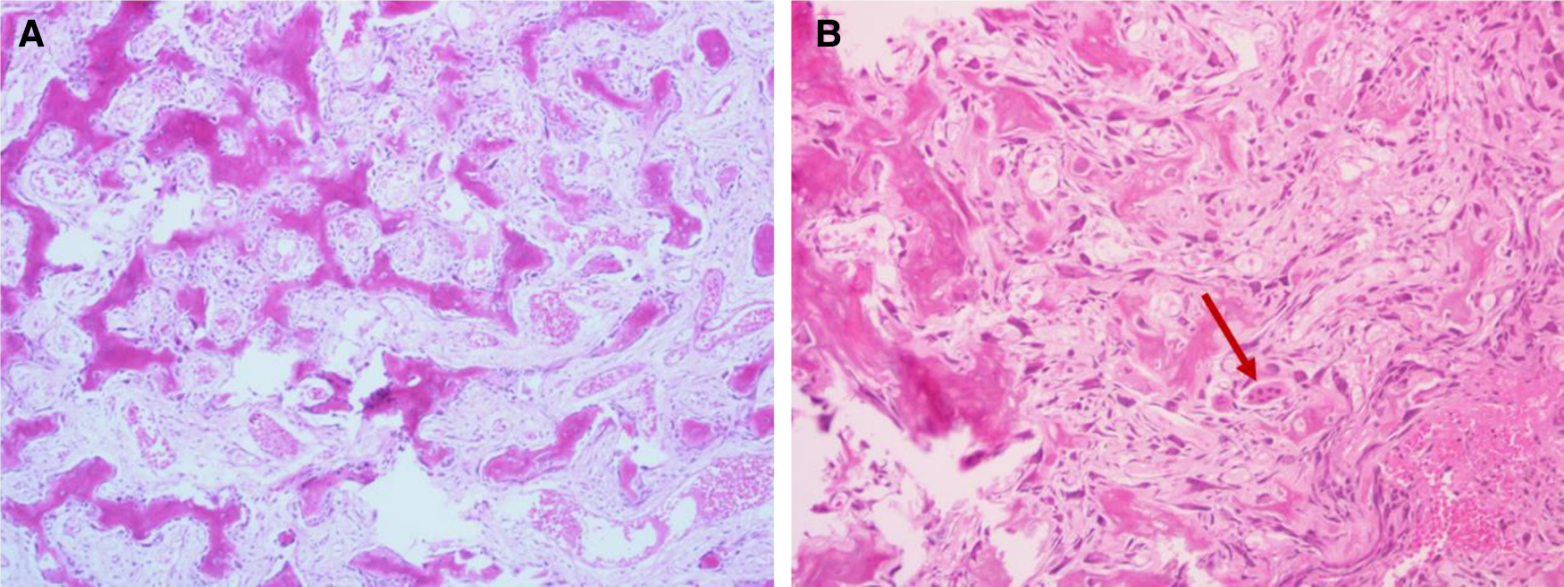
Histopathological features (H&E staining): (A) immature trabeculae rimmed by osteoblasts; (B) giant cells with multiple dense nuclei and cellular atypia can be found. H&E, Hematoxylin and eosin.

**Figure 4:**
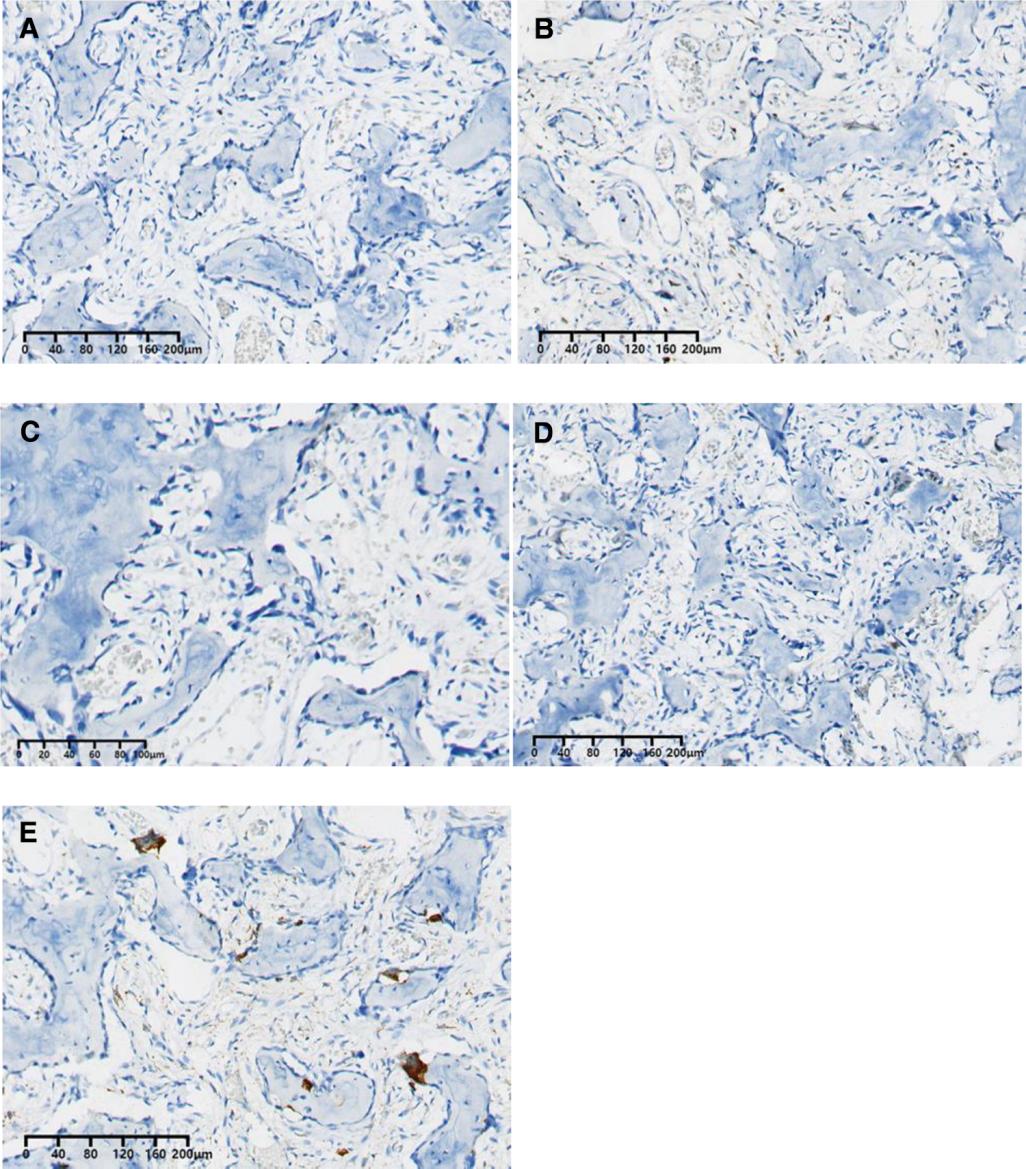
Immunohistopathological results: (A) Ki-67 (<5%); (B) PCNA (<5%); (C) SOX2 (+); (D) MDM2 (+); (E) CD68 scatteredly expression (+).

The histopathological examination was conducted after 7 days of decalcification in a 10% EDTA solution. It showed that the tumor was composed of haphazardly mineralized trabeculae of bone, osteoid rimmed by osteoblasts, and acellular vascular fibrous stroma (Figure 3A). There were giant cells with multiple dense nuclei, and cellular atypia can also be seen (Figure 3B), but no epithelioid osteoblast was seen within sight. Besides, the boundary of the tumor was not clear. The immunohistochemical results showed that both Ki-67 (Figure 4A) and PCNA (Figure 4B) labeling index were low (<5%), indicating that the proliferation capacity is relatively normal (11, 12). Positive expression of SOX2 (Figure 4C) and MDM2 (Figure 4D) indicated that the lesion was osteogenic (13–15). Scattered positive expression of CD68 (Figure 3E) proved the presence of multinucleated osteoclasts (16). Thus, a final diagnosis of conventional osteoblastoma was made with the aid of the immunohistochemical technique.

After one-month follow-up, postoperatively showed that the mouth opening range was normal without any pain or limitation, and the incision was quite concealed. Radiological examination (Figure 2E) also showed no difference compared with the immediate CBCT postoperatively (Figure 2D), which indicated that there were no signs of recurrence. Considering the huge trauma a second surgery will cause and the complete relief of his symptoms after recovering from the surgery, the patient chose to keep a regular follow-up to detect any recurrence instead of an immediate second surgery.

### Differentiation diagnosis

Due to the identical clinical and radiological features, it is hard to differentiate these bone lesions, including ossifying fibroma, osteoid osteoma, osteoblastoma, giant cell lesions of the jaws, and low-grade osteosarcoma.

Ossifying fibroma is composed of hyper-cellular fibroblastic stroma containing variable amounts of calcified structures. It is usually less vascular, more fibrous, and has less osteoblastic activity compared with osteoblastoma (17). Besides, the presence of abundant osteoblasts around the trabeculae also supports the diagnosis of osteoblastoma rather than ossifying fibroma (18). Osteoid osteoma is histologically identical to osteoblastoma. They are usually distinguished on the basis of size: osteoid osteoma is typically <1 cm and lesions >2 cm will be considered osteoblastoma (19–21). Osteoblastoma, on the other hand, can be locally aggressive, whereas osteoid osteoma lacks growth and invasiveness potential (1, 19). In this case, the main lesion was over 1 cm, and it affected the neighboring skull base radiologically, so it was more likely to be diagnosed as an osteoblastoma. Giant cell lesions of the jaws also show similar radiographic and clinical features to osteoblastoma (22). However, giant cell lesions of the jaws are always composed of fibrous connective tissue, irregularly distributed multinucleated giant cells, abundant blood vessels, a little bone-like tissue, and a large amount of hemosiderin in histopathological slices (23), so they can be distinguished accordingly.

A more significant differentiation to be made is between osteoblastoma and low-grade osteosarcoma. First, the latter one can be more aggressive and may have more severe symptoms clinically. Second, they can be distinguished by histopathological methods that show that hyperchromatic nuclei, mitosis, pleomorphic cells, and poorly circumscribed margins with infiltration of the host bone are more common in osteosarcoma (24, 25). Immunohistochemistry can also help to distinguish them; positive results for CDK4 and MDM2 support the diagnosis of osteosarcoma (26–29), for instance. Besides, osteoblastoma shows nuclear staining, whereas osteosarcoma shows cytoplasmic or membranous staining for β-catenin (30). Furthermore, osteoblastoma always shows strong and diffuse nuclear expression of FOS immunohistochemically, but it is usually negative in low-grade osteosarcoma (2, 31, 32). The expression of microRNA-210 is elevated in osteosarcoma when compared with osteoblastoma (33).

## Discussion

To our knowledge, there is only one case report of osteoblastoma at the temporal articular tubercle (34). And in our case, the lesion even affected the neighboring skull base, which showed bone swelling radiologically. The primary symptom of pain with mouth opening easily led to a diagnosis of the most common TMD and neglected the possibility of a benign tumor. This case reminds us to be careful with TMD diagnoses. Radiological examinations can sometimes be crucial in analyzing the location and features of the lesion. Moreover, histopathological examination is of great importance for the accurate diagnosis of osteoblastoma, and immunohistochemistry can additionally help with an auxiliary diagnosis, of which we used five antibodies, including Ki-67, PCNA, CD68, SOX2, and MDM2 in this case. Besides, according to recent findings, recurrent rearrangements of FOS and FOSB can be detected in osteoblastoma (2). Among them, FOS immunohistochemistry can be exploited as an auxiliary diagnostic tool for short-decalcified osteoblastoma tissue within 3 days (31).

As a matter of fact, gross total tumor resection is the standard treatment for osteoblastoma (35). Hence, a further surgery combined with the cooperation of the neurosurgery department is proposed for this patient. However, if the lesion was proximal to important structures, curettage would be the only feasible alternative to resection. Besides, carbon ion radiotherapy may also be a choice, which was reported to have good outcomes after 10 years of follow-up (36). However, because radiotherapy may cause malignant transformation of tumors (37, 38), it should be used prudently.

## Data Availability

The original contributions presented in the study are included in the article/Supplementary Material, further inquiries can be directed to the corresponding author.
